# Supplementary material for: Protein arginine methyltransferase 5 is essential for oncogene product EWSR1-ATF1-mediated gene transcription in clear cell sarcoma
Source: J Biol Chem. 2022 Aug 27;298(10):102434. doi: 10.1016/j.jbc.2022.102434 (PMC9513783; doi:10.1016/j.jbc.2022.102434)
Supplement: Supplementary [file mmc1.docx]

**Supplementary Information**

**Protein arginine methyltransferase 5 is essential for oncogene product EWSR1-ATF1-mediated gene transcription in clear cell sarcoma**

Bingbing X. Li,^1,^* Larry L. David, ^1, 2^ Lara E. Davis^2, 3^ and Xiangshu Xiao^1, 2,^ *

^1^Department of Chemical Physiology and Biochemistry, ^2^Knight Cancer Institute, ^3^Division of Hematology and Medical Oncology, Department of Medicine, Oregon Health & Science University, 3181 SW Sam Jackson Park Rd, Portland, OR97239, USA

**A**

**B**

**ASTDYSTYSQAAAQQGYSAYTAQPTQGYAQTTQAYGQQSYGTYGQPTDVSYTQAQTTATYGQTAYATSYGQPPTGYTTPTAPQAYSQPVQGYGTGAYDTTTATVTTTQASYAAQSAYGTQPAYPAYGQQPAATAPTRPQDGNKPTETSQPQSSTGGYNQPSLGYGQSNYSYPQVPGSYPMQPVTAPPSYPPTSYSSTQPTSYDQSSYSQQNTYGQPSSYGQQSSYGQQSSYGQQPPTSYPPQTGSYSQAPSQYSQQSSSYGQQSSFRQDHPSSMGVYGQESGGFSGPGENRSMSGPDNRGRGRGGFDRGGMSRGGRGGGRGGMGKILKDLSSEDTRGRKGDGENSGVSAAVTSMSVPTPIYQTSSGQYIAIAPNGALQLASPGTDGVQGLQTLTMTNSGSTQQGTTILQYAQTSDGQQILVPSNQVVVQTASGDMQTYQIRTTPSATSLPQTVVMTSPVTLTSQTTKTDDPQLKREIRLMKNREAARECRRKKKEYVKCLENRVAVLENQNKTLIEELKTLKDLYSNKSV**

**Figure S1**. (A) The surface rendering of AlphaFold-predicted structure of ATF1(66-271). (B) The EWSR1(2-325)-ATF1(66-271) fusion protein sequence. The EWSR1 portion is shown in green and the ATF1 portion is shown in purple.

**Figure S2**. The protein sequence of human PRMT5. The identified peptide sequences from Figure 2C are highlighted in red.

**Figure S3.** Differential binding modes of **GSK** compounds and **JNJ-64619178** to PRMT5. The left is the crystal structure of **EPZ015666** binding to PRMT5 (PDB: 4X61), where **EPZ015666** is an analog of **GSK591** and **GSK3326595**. This complex structure contains both **EPZ015666** and SAM in their respective binding pocket. **EPZ015666** binds at the protein substrate binding pocket. The right is the crystal structure of **JNJ-64619178** binding to PRMT5 (PDB: 6RLQ). **JNJ-64619178** occupies both the SAM pocket and protein substrate binding pocket.

**Figure S4.** Dose-response curves of PRMT5 inhibitors in a panel of CCSST cell lines: DTC-1 (A), SU-CCS-1 (B) and CCS292 (C) cells. The cells were treated with different concentrations of indicated drugs in 96-well plates for 3 days. Then the remaining viable cells were quantified by the MTT reagent. (D) DTC-1, SU-CCS-1 and CCS292 cells in 96-well plates were treated with different concentrations of **JNJ-64619178** for 6 days. Then the remaining viable cells were quantified by the MTT reagent.

**Figure S5**. Comparison of PRMT5 inhibitors in CCSST cells. (A) Chemical structures of PRMT5 inhibitors: **PF-0693999**, **EPZ015666**, **CMP5**, and **BRD0639**. Their apparent inhibition constants *K*_i_ are shown and from published references. The *K*_i_ for **CMP5** is unknown. (B) Summary of the GI_50_s (nM) of different PRMT5 inhibitors in 3 different CCSST cell lines. The cells were treated with different concentrations of the indicated compounds for 6 days. Then the remaining viable cells were quantified by the MTT reagent. The values represent the mean ± SD of at least 2 independent experiments performed in duplicates or triplicates.
